# Bibliometric Analysis of Accidental Oil Spills in Ice-Infested Waters

**DOI:** 10.3390/ijerph192215190

**Published:** 2022-11-17

**Authors:** Almat Kabyl, Ming Yang, Dhawal Shah, Arshad Ahmad

**Affiliations:** 1 Department of Chemical and Materials Engineering, School of Engineering and Digital Sciences, Nazarbayev University, Astana 01000, Kazakhstan; 2 Safety and Security Science Section, Faculty of Technology, Policy and Management, Delft University of Technology, Jaffalaan 5, 2628 BX Delft, The Netherlands; 3 Centre of Hydrogen Energy, Institute of Future Energy, UTM Johor Bahru, Universiti Teknologi Malaysia, Johor 81310, Malaysia; 4 Australian Maritime College, University of Tasmania, Launceston, TAS 7250, Australia

**Keywords:** oil spills, ice-infested waters, harsh environment, response, risk management

## Abstract

Oil spills are environmental pollution events that occur due to natural disasters or human activities, resulting in a liquid petroleum hydrocarbon release in the environment, especially into the marine ecosystem. Once oil spills happen, they cause detrimental consequences to the environment, living organisms, and humans. Although there are increasing oil and gas activities in the Arctic region, which is abundant with undiscovered oil and gas resources, the harsh environmental conditions of the region, such as the ice coverage, cold temperatures, long periods of darkness, and its remoteness, pose significant challenges to managing the risk of accidental oil spills in ice-infested waters. In this paper, a bibliometric analysis has been applied to study the global work on oil spill research in ice-infested waters. The paper aims to present an overview of the available oil spill response methods in ice-infested waters, identify the current trends of the research on oil spills in ice-infested waters, and determine the challenges with the future research directions based on the bibliometric analysis. The analysis includes a total number of 77 articles that have been published in this research field which were available in the Scopus database, involving 193 authors from 17 countries dating from 1960 to September 2022. During the bibliometric analysis, the top five most productive authors and countries as well as the most cited publications on oil spills in ice-infested waters have been identified; the authors’ cooperation network and the cooperation network between the countries in oil spills research in ice-infested waters have been created; a co-citation analysis and a terms analysis have been performed to identify the popular terms and topics. For future directions, it is recommended for researchers (1) to study real oil spills as much as possible to obtain a good overview through replication under different situations; (2) to develop a new technique for the careful examination and management of the potential risks; (3) to study oil separation from the recovered oil–ice mixture.

## 1. Introduction

Oil production and transportation entail the risk of accidental oil spills, which have detrimental consequences on the marine environment. Increasing the offshore oil and gas activities in the Arctic region shows that implemented available oil spill response strategies in the Arctic region is crucial. According to the U.S. Geological Survey (USGS) estimation of the undiscovered conventional oil and gas resources, the Arctic region has 90 billion barrels of undiscovered crude oil and 44 billion barrels of undiscovered natural gas liquids. Hence, the Arctic makes up 13% of the world’s undiscovered oil and 20% of natural gas liquids in the world. Around 84% of these oil and gas resources may occur in offshore areas [1]. The harsh environmental conditions of the Arctic region, such as the ice coverage, its remoteness, cold temperatures, and it having a long period of darkness, pose challenges to implementing efficient oil spill response plans. The ice presence in the Arctic Ocean affects the transport and fate of the oil spills as spilled oil can interact with ice in different ways. Namely, the oil can be on the ice surface, trapped between the cracks of broken ice, encapsulated within the ice, and it may stay there until it is melted, or the oil can be under the ice surface. The oil spreading process in ice-infested waters is slow, as it might be expected, due to the low temperature and the fact that the presence of ice restricts the spreading by herding the oil [2]. The duration and severity of the ice coverage also differ greatly depending on the location of it and the time of the year [3]. These factors affect the efficiency of the oil spill response options. Hence, selecting an effective oil spill response strategy in the Arctic environment depends on the time and location.

An oil spill is one type of environmental pollution event resulting from the release of liquid petroleum hydrocarbon into the environment (mainly, the marine environment) due to human activities or natural disasters. Oil spills contaminate water sources, harm marine life, threaten public health, and disrupt the economy [4]. The spills can occur in the different parts of the oil industry: during the exploration and production activities in the offshore platforms and wells and during the oil transportation by oil tankers and pipelines. Oil spills during its transportation by oil tankers or pipelines are the largest and the most common source of oil spills [5]. International Tanker Owners Pollution Federation (ITOPF) has classified oil spills by size into three groups: small (<7 tonnes or 50 bbls), medium (7–700 tonnes or 50–5000 bbls), and large (>700 tonnes or 50,000 bbls) [6]. Oil spill response and prevention techniques help to diminish the potential environmental consequences of various oil spill scenarios.

Although a great effort has been made to decrease the amount of spillage through better industry practices and regulatory and technological prevention procedures in recent years, the risk of occasional substantial spills is still present. Therefore, it is essential to manage the risks of accidental oil spills and be prepared for an oil spill response, including worst-case scenarios [5]. The core of the oil spills study is to understand the risk of the oil spills since it includes both the probability of them, which incorporates the spill causes, rates, prevention strategies, and the consequences of them, which involves the oil toxicity, transport and fate of oil, ecosystem, and organism impacts. The impact of oil spills differs depending on the volume of spilled oil, and the type and source of the oil, the location of the spill, the oil spill response technique, and the environmental conditions. Risk mitigation is the ultimate goal of the oil spill response and prevention techniques [7].

Oil spill prevention procedures and better industry practices are essential to reducing the oil spill risk. However, the best approach to contain and manage the spill once it happens is to respond to it instantly, following an established management strategy for the oil spill response. The management strategy should help to minimize the detrimental consequences of the spill for the environment, living organisms, and humans. It should also help to choose the economically sound response options for a particular oil spill case. Well-established international and national management strategies and contingency plans provide the response team with the necessary oil spill response information and help them to promptly contain and clean up the spill.

This paper discusses the oil spill response techniques that can be used during the cleanup of spilled oil in ice-infested waters. A review and a bibliometric analysis were used to study the global work on oil spill research in ice-infested waters, and these are presented. A bibliometric analysis is a quantitative review of a substantial amount of literature, which gives insights into a specific research field. It is used to determine the leading authors and the significant publications and evaluate the research and collaboration patterns of the authors, institutes, journals, and countries [8]. Additionally, a bibliometric analysis can help to identify the research directions, essential topics, and terms in a particular research field [9]. The paper aims to present an overview of the available oil spill response methods in ice-infested waters, identify the current trends in oil spill research in ice-infested waters, and determine the challenges with the future research directions based on the bibliometric analysis. The rest of the paper is organized as follows: Section 2 gives a brief overview of the environmental impacts of oil spills on the ecological systems in ice-infested waters. The oil types and oil spill response techniques are presented in Section 3. Section 4 is devoted to the bibliometric analysis, the results, and the discussion. Finally, the conclusion, challenges, and future directions are summed up in Section 5.

## 2. Environmental Impacts of Oil Spills on Ecological Systems in Ice-Infested Waters

The aquatic environment and life are interdependent and interrelated. The destruction of the physical environment destroys the lives of some living organisms inhabiting that area, such as algae, plants, invertebrates, fish, birds, and mammals, which may harm other kinds of organisms that are further up the food chain. An oil spill can harm aquatic life in several ways, such as through direct physical contact, toxic contamination, the destruction of habitats and food sources, and impacting reproduction.

*Direct physical contact.* Direct contact with oil results in eye and skin irritation. Animal fur loses its insulating properties after it comes in contact with oil. As a result, it can lead to the risk of the animal freezing to death.

*Toxic contamination.* Oil particles can damage the central nervous system, liver, and lungs of the animals. Additionally, the risk of ingesting oil is high, leading to cell damage in the intestinal tract, stomach disorders, and a reduction of the animal’s capability to eat and digest food.

*Destruction of habitats and food sources.* An oil spill can also affect the species that are not in direct physical contact with oil. If a prey population is destroyed due to an oil spill, it leaves the predators with no food resources [4].

*Reproduction.* An animal that comes in contact with oil can suffer from impaired reproduction. Moreover, juveniles and eggs are especially vulnerable. Even contact with minimal amount of oil can be fatally toxic to the embryos [10].

In this event, strict marine oil spill laws account for the oil spill preventive regulations. The amount of fines and penalties should be high enough to exceed the amount of money that is needed to avoid and minimize the oil spill risk, including but not limited to the installing prevention devices, implementing better training, enhancing the safety practices, etc. The objective of these laws is to serve as deterrents for future accidents and impose a penalty on the responsible sides who have already committed an offense. Table 1 shows marine oil spill laws in certain countries and marine oil spill fines and penalties.

## 3. Oil Type and Oil Spill Response Techniques

Although the characteristics of each oil are unique depending on the geographical location, toxicity, and persistence of it, it is helpful to categorize them into four general groups (as shown in Table 2). These groups are not only based on the specific gravity of the oil, but they also include the concentrations of: more toxic and less persistent compounds—aromatics; less toxic but highly persistent compounds—heavier components.

*Group I—volatile distillates.* Fuels including kerosene, jet fuel, gasoline, and crude condensate are examples of volatile distillates that are highly toxic, but they evaporate rather quickly. This category includes hydrocarbon fractions that distill at least 50% of its volume at 340 °C and at least 95% of its volume at 370 °C.

*Group II—light fuels.* Refined petroleum products and crude oils which are moderately toxic and have a low persistency level are included in the category of light fuels. As opposed to the volatile distillates, these oils such as gas oil, diesel fuel, hydraulic oil, and light crude oil do not evaporate as quickly. Refined petroleum products and crude oils with a specific gravity of less than 0.85 (American Petroleum Institute (API°) > 35.0) are included in this category.

*Group III—medium fuels.* The majority of crude oils, synthetic crudes, intermediate fuel oil, and lube oil fall within the category of medium oils as they are moderately persistent and moderately toxic. The specific gravity of Group III is between 0.85 and 0.95 (API° ≤ 35.0 and >17.5).

*Group IV—heavy fuels.* Heavy fuels are defined as very persistent, but they are less toxic petroleum products and crude oils. Heavy fuel oil and heavy crude oils are included in this category. Group IV oils have a specific gravity of between 0.95 and 1.0 (API° between 17.5 and 10.5) [7].

*Oil spill response techniques.* In the oil spill response process, the response effectiveness relies on one properly selecting a response option. There are several effective techniques that are available for an oil spill response, such as:

- Mechanical recovery;
- The usage of dispersants;
- In situ burning.

Each response option has particular advantages and limitations, which can be crucial when one is selecting a response option. Additionally, the effectiveness and feasibility of the response options in ice-infested waters depend on the seasonal ice. In different seasons, various oil spill advantages and disadvantages arise:

- In the open water summer period, the oil spill response is the same (apart from the remoteness of the region) as it is in temperate waters;
- During ice growth and freeze-up, there is limited site access and drifting ice which impose limits on the feasible response methods;
- In the middle of the winter, when there is an extremely cold environment and a long dark period, a stable ice cover restrains the oil within the bounds of a relatively compacted area, and this presents a safe working platform for an oil spill response in the case of fast ice being present. In the case of drifting ice, it is the opposite. It can transport entrapped irregular oil slicks to areas that are far beyond the spill site and contaminate a large region [11,12];
- During the final ice melting and thaw, the oil spill response is more limited in moving ice packs because of the ice pack’s changing nature and the necessity for ice-strengthened vessels.

### 3.1. Mechanical Recovery

The main objective of mechanical containment procedure is to confine the spilled oil and restrict it from spreading by keeping it within the confines of a boom and recovering the oil from the marine environment to dispose of it. The removal of oil from the sea surface decreases the environmental and socioeconomic damages. The mechanical recovery’s strong point is that the spilled oil can be removed from the surface of the water, thus preventing it from drifting to the shore at the end. However, it is a slow process, and the oil can disseminate faster than it can be recovered, which is a disadvantage of the method.

Firstly, the physical properties of the oil should be identified when one is addressing oil spills, namely, its pour point. If it is 5–10° C above the water temperature, then the oil will most likely be solid. Collection devices and nets may be needed for recovery. If the pour point is lower than the temperature of the water and if the wind and currents conditions allow it, then skimmers and booms may be appropriate to use [12].

*Booms* are used to limit the spreading of the oil to decrease the probability of shoreline pollution and other resources and concentrate oil in thicker layers to make the recovery process more manageable. Using a floating boom to restrain the spilled oil is not practical if the area is covered with ice or if the oil is encapsulated within the ice. If the ice coverage is higher than 6/10th of the size of the area, the ice itself can be a natural containment barrier [13].

A skimmer is a device that has been designed to recover spilled oil from the surface of the water. Brush skimmers and the oleophilic rope are the most suitable skimmers for ice-infested waters because other skimmer types rapidly become clogged with smaller ice pieces. Even the negligible ice concentrations impact most of the skimmer systems’ performances through bridging and plugging. Oleophilic skimmers collect a minimal amount of water when they are compared to the recovered oil amount. They work effectively on relatively thin oil slicks and are less vulnerable to debris and ice than other skimmer types are [14]. Table 3 briefly summarizes the skimmer’s efficiency. 

### 3.2. Usage of Dispersants

Using dispersants moves the oil from the sea surface to the water column. This way, it does not allow the oil to drift and contaminate the shoreline in the end. When dispersants are used, the surfactants in the dispersant decrease the interfacial tension between the seawater and the oil. This allows the wind and waves to break down larger oil droplets into smaller droplets, which can be diluted into the water column. Eventually, tiny droplets in the water column can be biodegraded by microorganisms [16]. The recommended rate of the dispersant-to-oil ratio (DOR) is 1:25. According to Lewis and Daling [17], the dispersants’ efficiency in the Arctic environment can be reduced due to there being colder temperatures and ice presence in the sea. The presence of the ice coverage of more than 9/10th of the size of the area makes the application of the dispersants impossible since the ice cover encapsulates the oil, and the energy that it would take to break the spilled oil into droplets is not sufficient. When the ice coverage is less than 3/10th of the size of the area, the dispersants’ efficiency is the same as it would be in an open water environment. The efficiency of the dispersants when the ice coverage is between 3/10th and 8/10th of the size of the area (without additional energy application) is somewhat uncertain [18]. Additionally, the oil viscosity is another major factor that has an impact on the dispersibility of the oil. Most studies concluded that there is a viscosity limit that, above which oil is not dispersible. This limit depends on the oil type, but the dispersibility decreases with an increasing viscosity. A general principle is that the oil is dispersible when the viscosity is less than 2000 cP (centipoise), and the oil is not dispersible when the oil viscosity is above 10,000 cP (centipoise) [19,20]. Table 4 gives a summary of these operating limits. 

The characteristics of the Arctic environment, namely, the remoteness of it, the long darkness periods and the low temperatures during wintertime, and the presence of ice, pose difficulties to the dispersants usage and other oil spill response methods. However, the results of studies over the last 40 years show that dispersant usage in the Arctic environment is a feasible oil spill response option.

### 3.3. In Situ Burning

In situ burning is an oil spill response option involving ignition and controlled oil combustion. The first in situ burning experiments were carried out in the Canadian Beaufort Sea in the 1970s [21]. As a result of a successful experiment series which was conducted in the 1970–1980s, in situ burning was recognized as an efficient oil spill response option in ice-infested waters. According to Brandvik et al. [22], the effectiveness of in situ burning in field tests is in the range between 50% and 90% (in ice coverages of 7/10th–9/10th of the size of the area). SL Ross Environmental Research identified API gravity as a single good predictor of in situ burning efficiency success. It developed guidelines for in situ burning as a function of API gravity. Light oils with 35° or a higher API gravity burned easily, whereas heavy oils with 20° or a lower API gravity were hard to ignite, or they were not ignitable. Table 5 shows the efficiency of in situ burning as a function of API gravity [19].

Some of the challenges of in situ burning are to sustain enough oil thickness to maintain a burn. The minimum thickness that can be ignited for fresh crude oil is 1 mm, whereas, for aged crude oil, it is 2–5 mm [21]. In situ burning is usually used on open water with a mechanical recovery technique. In many cases, fire-resistant booms gather the oil in a restricted area and concentrate to provide a thick layer to burn. It can be harder to ignite oil at low temperatures, but it will continue despite the ambient temperature once it is ignited and the burning begins. The wind and current conditions can influence the in situ burning efficiency, but ice is also an essential factor. If the ice coverage exceeds 7/10th of the size of the area, then the mechanical recovery options to not need to be applied to in situ burning since the ice coverage can serve as a natural containment barrier. If the ice coverage is less than 3/10th of the size of the area, then open water in situ burning can be an appropriate option together with the usage of the fire-resistant boom [23]. Ice concentrations that are between 3/10th and 7/10th of the size of the area are considered to be the hardest to deal with in terms of in situ burning since the ice content is high enough to decrease the efficiency of mechanical recovery, but it is low enough to be a natural containment barrier [21]. The possibility of using in situ burning as a response option reduces with the oil weathering and time. Using in situ burning within 72 h after a spill is practical. For thick or heavy oils, in situ burning might be suitable only within 1–2 h after a spill [19]. In general, in situ burning can be the preferred oil spill response option if it is unsafe to work on the ice. At the end of the burning period, 5% of the initial oil volume, mainly the high-density terry residue, will remain. The in situ burning residue may sink or float depending on the type of oil and the extent of the burning.

### 3.4. Modelling of Oil Spills in Ice-Infested Waters

Oil spill modeling in ice-infested waters is based on oil spill models in open waters with some adjustments, which rely on the input parameters updates using experiment results in an icy environment. Some software is available for oil spill modeling in ice-infested waters [24]. For instance, the model that as developed by Ovsienko, Zatsepa, and Ivchenko [25] forecasts the oil spreading between fixed ice floes, whereas de Silva and Yamaguchi [26] developed a simulation model to predict the spilled oil behavior in ice-covered waters. As the oil weathering processes greatly depend on the oil type and environmental conditions, the SINTEF developed and integrated an ice module into the SINTEF Oil Weathering Model and the OSCAR (Oil Spill Contingency and Response) model. The models were updated with the results of mesoscale laboratory tests and full-scale field experiments in ice-infested waters to model and predict the oil weathering properties [27,28]. Additionally, the models such as the blowout and spill occurrence model (BLOSOM), SIMAP/OILMAP, the general NOAA operational modeling environment (GNOME) can be updated and adjusted to ice-infested waters. Table 6 provides an overview of various oil spill modelling software. 

There are limited research studies on developing integrated and effective decision-making frameworks to support oil spill responses, especially in ice-infested waters, in an environmentally friendly and cost-efficient manner. Most of the oil spill literature focuses on studying the transport and fate of oil spills, and these studies are usually considered to be separately from the oil spill response studies [30]. Gkonis et al. [31] proposed a model-based tactical oil spill response model, which accounts for the weathering process as an important factor in the decision-making process of an oil spill. Decision making is a complex process that enables decision makers to improve their decision-making effectiveness by investigating and simulating alternative decision scenarios. During the decision-making process of the oil spill response options, a wide range of factors should be considered, such as the environmental sensitivity, economic importance, the cost of potential response techniques, and their relative importance should be assessed by the decision maker [32]. To better the support oil spill response and decrease the damage that an oil spill may cause, there is a need to develop new decision-making approaches and a framework to determine the most suitable oil spill response option for a specific case of ice-infested waters which takes into account the risks that are associated with the oil spill response strategies.

## 4. Bibliometric Analysis: Results and Discussion

The data that were to conduct the analysis were obtained from Scopus on 1 September 2022. The term “oil spills in ice-infested waters” was used as the search topic to find all of the articles from 1960 to 2022 that contained these words in the article title, abstract, or keywords. In total, 77 publications were found, and all of the publications were used for the further analysis, namely, to determine the publication growth trends, the cooperation between the authors and countries, the co-citation patterns between the authors, and the highly used terms in the oil spill publications in ice-infested waters.

Various software is available to conduct a scientometric analysis and visualize the outputs [33]. In this study, VOSviewer 1.6.15, which was developed by Van Eck and Waltman (VOSviewer: Leiden, The Netherlands) [34], was used to perform the bibliometric analysis and visualize the networks of the authors, countries, co-citations, and terms. This software analyzes the relatedness and similarities between the publications in terms of the keywords co-occurrence, co-authorship, co-citation of references, and common terms in the titles and abstracts, which are recognized through text mining techniques. In general, the visualization output can be interpreted in the following way: the circle size and label font illustrate the number of occurrences, the distance between the circles shows the similarity and relatedness between the publications, and the colors depict the clusters/different groups with the time information, and the *x*-axis and the *y*-axis have no meaning [35].

**Figure 1:**
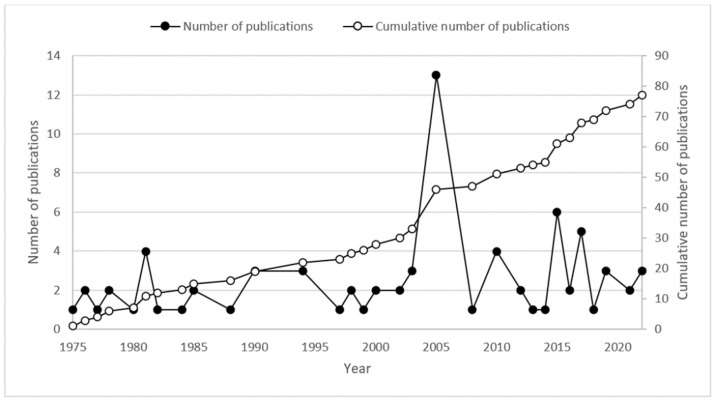
Number of publications and cumulative number of publications on oil spills in ice-infested waters according to the year.

As shown in Figure 1, the global publication outputs on oil spills in ice-infested waters are limited, with there being 77 publications. There were less than 30 publications on the topic until 2000. In 2005, the highest peak of publications was observed with 13 publications, after which the number of new publications fluctuated. There were six recent publications in 2015, and there were another five articles in 2017. From the cumulative number of publications, it can be concluded that the scientific attention on the topic is insufficient, i.e., not much work has been conducted. Hence, oil spill research in ice-infested waters has great potential for future developments. There is a considerable opportunity for the use and implementation of the risk analysis and decision support frameworks, which can be a major research topic in the area.

A total number of 193 authors have contributed to the research area. The vast majority of the authors (78.2%, *n* = 151/193) have one publication on oil spills in ice-infested waters, and only 13 authors (6.7%, *n* = 13/193) have published three or more papers. Table 7 shows the top five most productive authors who have published on oil spills in ice-infested waters. According to the Scopus data, Hans V. Jensen (NOFO, Norway) is the most productive author on oil spills in ice-infested waters, with a total of seven publications. Mervin F. Fingas, Janne Fritt-Rasmussen, S. Venkatesh, and Ali S. Rangwala have four publications each, and factors such as the average citations per publication and the number of publications as the first author were taken into account.

**Figure 2:**
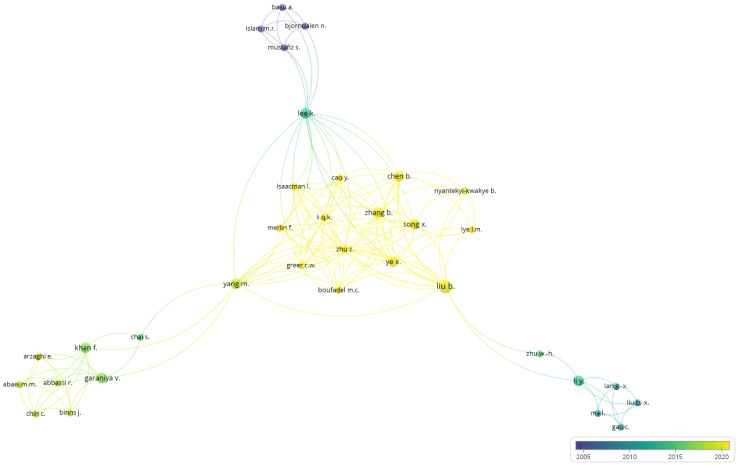
Authors’ cooperation network in oil spills research in ice-infested waters.

Scientific cooperation is a measure and indicator of the development of a discipline. The output of scientific collaboration is co-authored publications. The cooperation degree is calculated by dividing the total number of authors by the total number of papers. In this analysis, the cooperation degree of the authors is 2.5, which represents the average number of authors per article. The authors’ cooperation pattern was analyzed using VOSviewer. The largest set of connected authors consisted of 34 researchers, and other authors who were not connected with the other researchers were not included in the network. The authors’ cooperation network in oil spill research in ice-infested waters is depicted in Figure 2. The circles’ size illustrates the number of publications. The lines between the authors show the strength of collaboration between them. The colors represent the author’s average publication year on the topic.

**Figure 3:**
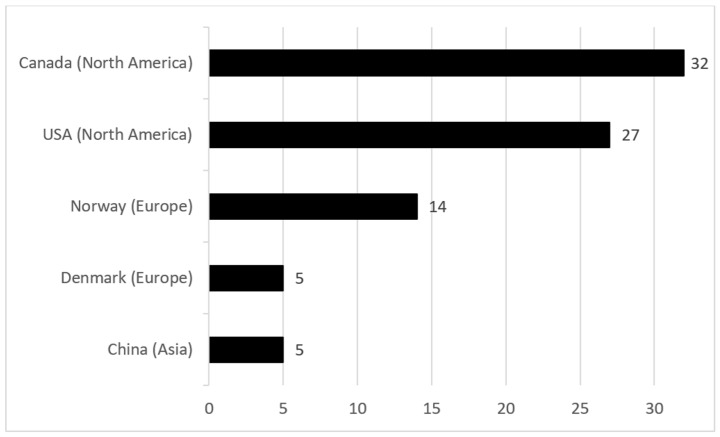
Top 5 most productive countries in oil spills research in ice-infested waters.

The broadness of the geographical coverage of the research collaborations is a measure of recognition and versatility of the research topic [36]. The researchers in oil spills research in ice-infested waters are from 17 different countries. Out of seventeen countries, thirteen of them are located in Europe (Norway, Denmark, and others), two of them are located in North America (Canada and USA), one of them is in Asia (China), and one of them is in Oceania (Australia). Figure 3 illustrates the top five most productive countries in oil spill research in ice-infested waters and the contribution of each country to the field. The analysis of the geographical distribution of the literature shows that twelve countries (70.6%) have published fewer than five articles, two countries have published five articles, and only three countries (17.6%) have published more than 10 articles.

**Figure 4:**
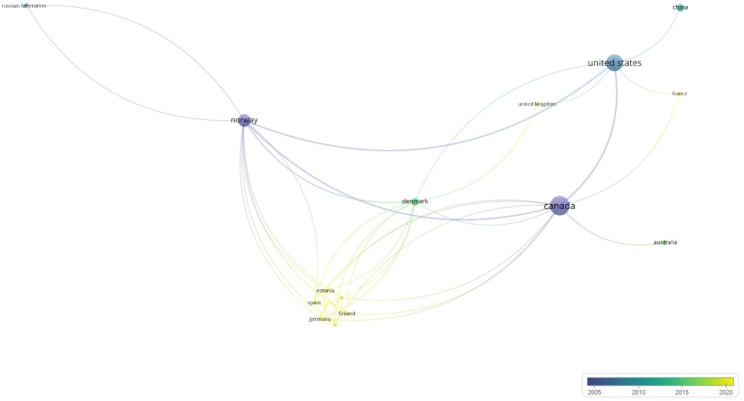
Cooperation network between countries in oil spills research in ice-infested waters.

The cooperation network between the countries in oil spill research in ice-infested waters is depicted in Figure 4. The countries that are not connected with other countries are not included in the network. The size of the circles indicates the number of publications, the thickness of lines indicates the collaboration strength, and the colors represent the average year of the publications.

*Cited analysis.* The cited analysis examines the number of citations that each publication has received. In total, 77 publications received 397 citations, with an average of 5.2 citations per publication. Half of the publications received zero citations, 15.6% pf the, (*n* = 12/77) received 10 or more citations, and only one publication was cited more than 50 times. Table 8 shows the top five most cited publications on oil spills in ice-infested waters. The most cited article is “Review of the behavior of oil in freezing environments” by Fingas, which has been cited 94 times (according to Scopus data as of September 2022). Among the top five most cited publications on the oil spill in ice-infested waters, Canada is best represented country with the two most cited publications. Two out of five most cited papers were published in Marine Pollution Bulletin, whereas the others were published in Process Safety and Environmental Protection, Journal of Environmental Management, and Atmosphere-Ocean. The publications in highly reputable journals indicate the importance of the field, i.e., the journals pay attention to the issues that are raised in the research domain. Therefore, future potential hot topics may be in the direction of oil spill research in ice-infested waters.

**Figure 5:**
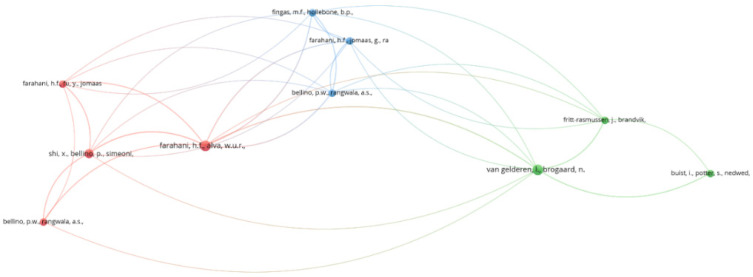
Co-citation analysis of cited authors used in publications on the oil spills in ice-infested waters.

*Co-citation analysis.* The co-citation analysis shows the interaction between two papers and reviews the publications that have been cited together [42,43]. To be included in the co-citation analysis, each article has to be cited at least two times. Thirty-two articles met the criteria and out of the thirty-two articles, the largest set of connected articles consisted of ten articles. The result of the co-citation analysis is depicted in Figure 5. The circle size represents the number of citations that were received, the distance between the circles indicates how close the publications are, and the colors of the clusters represent the similarity of the publication topics.

**Figure 6:**
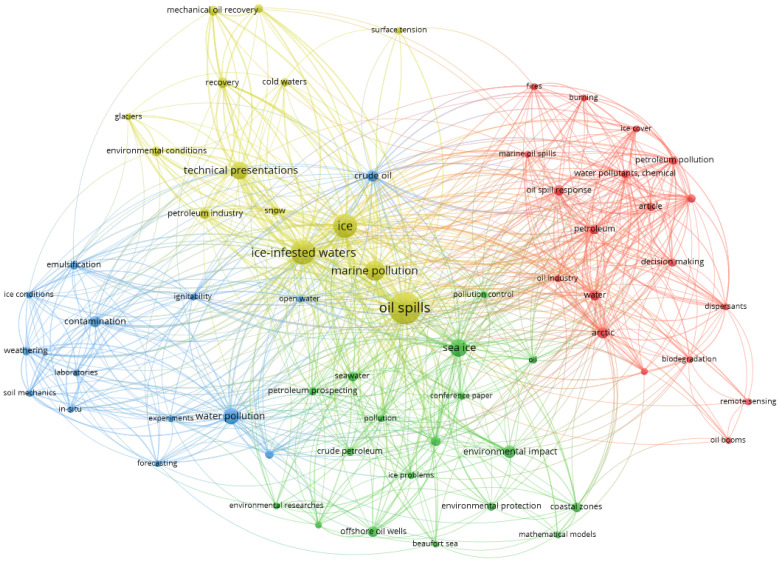
Analysis of terms in oil spills publications in ice-infested waters.

*Terms analysis.* To identify the research trends in oil spill research in ice-infested waters, a terms analysis can be used. During the analysis, the terms used in the article title and abstract were analyzed with the application of the text mining technique. As a result, a terms heat map was generated. VOSviewer analyzed 656 terms that were used in 77 papers using the binary counting of the terms, i.e., each term was counted only once per article. The terms that occurred at least three times were used in the network, and 66 terms met the threshold. The results of the terms analysis are shown in Figure 6. The circles’ size shows the number of occurrences of the term, the distance between the terms indicates their relatedness (if the words occurred together), and the colors represent the different clusters. There are four clear clusters, and the most common keyword in each cluster are oil spills (yellow), arctic (red), water pollution (blue), and sea ice (green).

**Figure 7:**
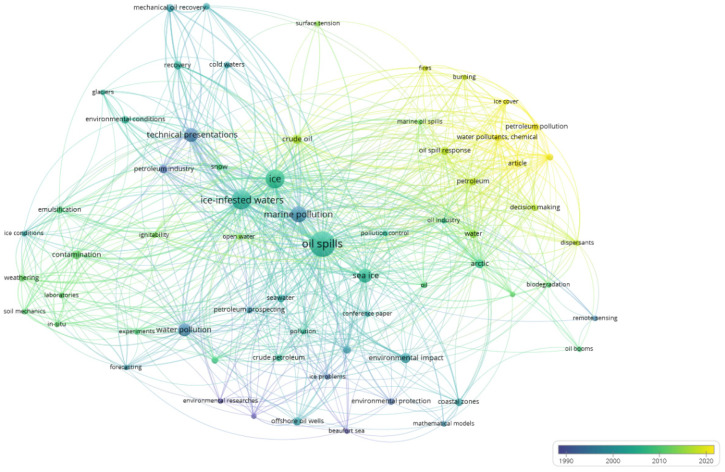
Analysis of terms in oil spills publications in ice-infested waters with time information.

**Figure 8:**
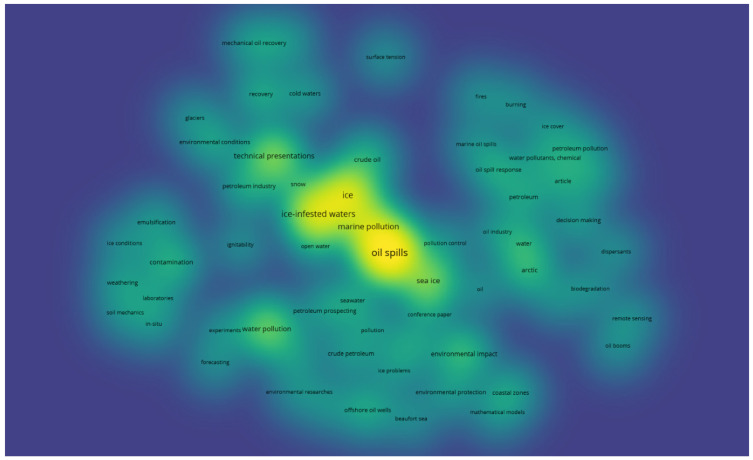
Terms heat map of oil spills publications in ice-infested waters.

Figure 7 represents a terms analysis of the oil spill publications in ice-infested waters with the time information. The color of each term shows the average year of the publication in which the term was used. In the terms analysis, the terms from the 1990s are grouped with blue color, the terms that were used in the 2010s are marked with a green, whereas the terms that were commonly used in the 2020s are shown with yellow color. The term “ice-infested waters” is labeled as green, showing that the publications on the topic of oil spills in ice-infested waters are relatively new when they are compared to the oil spills in temperate regions. The intersection of the recent terms lies in the decision-making process in the area of oil spill response in ice-infested waters. However, this area is not well investigated as the sizes of the circles are not big enough. The terms heat map of the oil spill publications in ice-infested waters is depicted in Figure 8, which provides complementary information on the research field in addition to Figure 6 and Figure 7.

The identification of the popular terms and topics is the key to understanding the development of a research field. As a research area develops, the researchers conduct intensive work and they intersect in essential research directions, leading to the maturity and growth of a certain area. Therefore, pinpointing the hotspots of a research domain helps to track the trends and better understand the field. Figure 8 shows the most heated terms, which are general terms such as oil spills, ice, marine pollution, and ice-infested waters. It can be seen that other words are not widely used, showing that the research on the topic is scarce. The font size of the terms represents the relative advancement and significance of a term.

## 5. Conclusions

The abundance of undiscovered oil and gas resources in the Arctic leads to increased oil and gas activities in this region, and consequently, the risk of potential oil spills is also increasing. This paper presented a bibliometric analysis of the oil spill research in ice-infested waters from 1960 to September 2022. The analysis provides insights into the particular research area, and it helps to determine the essential terms and research directions, significant publications, leading authors, and collaboration patterns of authors. It was observed that the number of publications on oil spills in ice-infested waters is limited, i.e., the scientific attention on the topic is not sufficient. Hence, there is a potential for future developments and a considerable opportunity for the use and implementation of risk analysis, decision support frameworks, which can be a major research topic in the area. The intersection of the recently used terms lies in the decision-making process in the area of oil spill response in ice-infested waters. The analysis includes 77 publications involving 193 authors from 17 countries. During the bibliometric analysis, the following patterns have been identified:

- The vast majority of the authors (78.2%) have published one paper on oil spills in ice-infested waters, and only 6.7% of the authors have published three or more articles;
- Out of seventeen countries, twelve countries (70.6%) have published fewer than five articles, two countries have published five articles, and only three countries (17.6%) have published more than ten articles;
- Half of the publications did not receive any citations, 15.6% of them received ten or more citations, and only one publication was cited more than 50 times.
- Through the analysis, the most influential contributors have been identified:
- The comparatively high-yielding author Hans V. Jensen (NOFO, Norway) contributed to the topic the most with a total of seven publications;
- “Review of behavior of oil in freezing environments” by Fingas and Hollebone [37] is the most cited article, which has received 94 citations;
- Canada, the USA, and Norway are the top three countries that have contributed to oil spill publications in ice-infested waters with 32, 27, and 14 publications, respectively.

Previous studies have primarily aimed to understand the physical environment, create a model that can predict the short-term movement, transport, and fate of spilled oil in ice-infested waters, investigate the oil spill response methods, and evaluate their effectiveness in ice-infested waters. The challenges that are posed by the Arctic are associated with harsh its environmental conditions, remoteness, infrastructure limitation or its absence, and the sensitivity of the ecosystem, which cause significant limitations on the effectiveness and operational feasibility of a clean-up. Since the field environment differs across locations and over time, it is recommended to study real oil spills as much as possible to obtain a good overview through its replication under different situations. It is also recommended to develop a new technique for the careful examination and management of the potential risks before engaging in large-scale oil spill response operations in ice-infested waters. This way, safe and sustainable operations can be ensured. Further studies related to oil separation from the recovered oil–ice mixture are also suggested to be conducted.

## Figures and Tables

**Table 1:** Marine oil spill fines and penalties [7].

Country	Marine Oil Spill Law	Fines and Penalties
Canada	Canada Shipping Act Migratory Bird Act (MBA) Fishing Act Canadian Environmental Protection Act	*Minor offenses:* To CAD 250,000 [USD 168,175] and/or 6 months prison; *Major offenses:* To CAD 1 million [USD 672,700] and/or 3 years prison; *Under MBA:* To CAD 520,000 [USD 350,000].
Japan	Marine Pollution Prevention Law	*Intentional spill:* To JPY 10,000,000 [USD 93,370]; *Unintentional but at fault:* To JPY 5,000,000 [USD 46,685].
UK	Merchant Shipping (Prevention of Oil Pollution) Regulations of 1996	*Magistrate’s Court:* To GBP 250,000 [USD 409,641]; *Crown Court (most serious offenses):* unlimited fines.
US	Oil Pollution Act of 1990 Federal Water Pollution Control Act Comprehensive Environmental Response, Compensation, and Liability Act of 1980	*Class I civil penalties:* To USD 10,000–25,000 maximum; *Class II civil penalties:* To USD 10,000 per day to USD 125,000 maximum; *Judicial civil penalties:* To USD 25,000 per day or USD 1000/bbl (USD 7000/t) spilled; *Gross negligence:* USD 100,000 minimum to USD 3000/bbl (USD 21,000/t) spilled; Additionally, fines and penalties by state.

**Table 2:** Oil type classifications [7].

Oil Type	Persistency	Toxicity	Group Characteristics	Examples
Group I—volatile distillates	Non-persistent	Highly toxic	Distill between 340 °C (at least 50% by volume) and 370 °C (95% by volume)	Jet fuel, kerosene, gasoline, crude condensate
Group II—light fuels	Low persistent level	Moderately toxic	API gravity > 35.0	Diesel fuel, gas oil, light crude oil, hydraulic oil
Group III—medium fuels	Medium persistent level	Moderately toxic	API gravity ≤ 35.0 and >17.5	Most crude oils, intermediate fuel oil, lube oil, synthetic crudes
Group IV—heavy fuels	High persistent level	Less toxic	API gravity ≤ 17.5 and >10.0	Heavy fuel oil, heavy crude oils

**Table 3:** The efficiency of skimmers [15].

Skimmer Type	Efficiency Notes
Oleophilic	
Brush	- High efficiency (>90%) in viscous oils; - Not efficient in refined products.
Rope	- Efficient in light, refined products (80–99%); - Reduced efficiency in heavier oil.

**Table 4:** Operating limits of dispersants [19,20].

Conditions		Dispersible (>70%)	Reduced Dispersibility (5–70%)	Not Dispersible (<5%)
Wind speed, m/s		<13	≥13 to ≤15	>15
Ice cover, 10ths		<5/10	5/10 to 9/10	>9/10
Viscosity, cP	Alaska North Shore (ANS) crude	<1000	1000–10,000	>10,000
	Bonnie Light crude	<500	500–2000	>2000

**Table 5:** The efficiency of in situ burning as a function of API gravity [19].

Fuel Type	Example	API Gravity	Burning Efficiency
Refined	Diesel fuel oil	37.2	90–98
Light crude	S. Louisiana crude	38	85–98
Medium crude	West Delta 143	30	80–95
Heavy crude	West Delta 30	23	75–90
	Santa Ynez	17	Not ignitable
	Bitumen	<10	13

**Table 6:** Comparative information of different oil spill models [29].

Model	Restrictions	Movement	Applications	Dimensions	Owner
OSCAR	Proprietary	Hydrodynamic models	Surface or subsurface	3D	SINTEF
BLOSOM	Open source	Various hydrodynamic models	Surface, subsurface	3D + time	Department of Energy, National Energy Technology Lab
SIMAP/OILMAP	Proprietary	Various hydrodynamic models	Surface, subsurface	3D, 2D + time	RPS Applied Science Associates
GNOME	None	СATS hydrodynamic model	Surface (subsurface module is available)	2D (3D with subsurface module)	National Oceanic and Atmospheric Administration

**Table 7:** Top 5 most productive authors publishing on oil spills in ice-infested waters.

No.	Author’s Name	Institution, Country	Number of Publications	Average Citations per Publication	Number of Publications as the First Author
1	Hans V. Jensen	NOFO, Stavanger, Norway	7	2.71	3
2	Mervin F. Fingas	University of Alberta, Edmonton, Canada	4	24.5	3
3	Janne Fritt-Rasmussen	Aarhus University, Aarhus, Denmark	4	15.25	0
4	S. Venkatesh	Environment Canada, Gatineau, Canada	4	13.25	2
5	Ali S. Rangwala	Worcester Polytechnic Institute, Worcester, United States	4	6.75	0

**Table 8:** Top 5 most cited publications on the oil spills in ice-infested waters.

No.	Title	Author(s)	Country (Affiliation of the First Author)	Journal Name and Impact Factor	Year	Citations Received	Average Citation per Year
1	Review of behavior of oil in freezing environments [37]	Fingas, M.F., Hollebone, B.P.	Canada	Marine Pollution Bulletin (7.001)	2003	94	4.95
2	Modelling the behavior of oil spills in ice-infested waters [38]	Venkatesh, S., El-Tahan, H., Comfort, G., Abdelnour, R.	Canada	Atmosphere—Ocean (2.058)	1990	47	1.47
3	A hierarchical Bayesian approach to modelling fate and transport of oil released from subsea pipelines [39]	Arzaghi, E., Abaei, M.M., Abbassi, R., (…), Chin, C., Khan, F.	Australia	Process Safety and Environmental Protection (7.926)	2018	30	7.5
4	Effectiveness of a chemical herder in association with in situ burning of oil spills in ice-infested water [40]	van Gelderen, L., Fritt-Rasmussen, J., Jomaas, G.	Denmark	Marine Pollution Bulletin (7.001)	2017	23	4.6
5	Scale-up considerations for surface collecting agent assisted in situ burn crude oil spill response experiments in the Arctic: Laboratory to field-scale investigations [41]	Bullock, R.J., Aggarwal, S., Perkins, R.A., Schnabel, W.	USA	Journal of Environmental Management (8.910)	2017	18	3.6

## Data Availability

The data to conduct the analysis were obtained from Scopus on 1 September 2022. https://www.scopus.com/results/results.uri?sort=plf-f&src=s&st1=oil+spills+in+ice-infested+waters&sid=f81b5e06b9eec39f2b47916c3c367175&sot=b&sdt=b&sl=48&s=TITLE-ABS-KEY%28oil+spills+in+ice-infested+waters%29&origin=searchbasic&editSaveSearch=&yearFrom=Before+1960&yearTo=Present (accessed on 18 October 2022).
